# Supplementary material for: Complexome profiling of the Chlamydomonas psb28 mutant reveals TEF5 as an early PSII assembly factor
Source: Plant Cell. 2025 Mar 17;37(6):koaf055. doi: 10.1093/plcell/koaf055 (PMC12164590; doi:10.1093/plcell/koaf055)
Supplement: koaf055_Supplementary_Data [file koaf055_supplementary_data.zip › psb28_interactive.zip!/plots/intensity/qualityPlots/peptides/CGL129_Cre05.g233950.t1.2.html]

### Cre05.g233950.t1.2

Trivial name: CGL129  
SeqCoverage [median]: 32.60  
Mass: 15.64 kDa  
  
 

| Sequence | MissedCleavage | Mass [Da] | wtIntensity | muIntensity | Score |
| --- | --- | --- | --- | --- | --- |
| AEQSSSTETTSFDSEK | 0 | 1732.73 | 12480.63 | 14400.03 | 274.92 |
| DIGDLSK | 0 | 746.38 | 109204.84 | 156507.93 | 108.70 |
| DIGDLSKK | 1 | 874.48 | 4412.05 | 10146.74 | 95.74 |
| DLQEKWDAVDNK | 1 | 1459.69 | 2395.20 | 2765.02 | 101.39 |
| EELMKDIGDLSK | 1 | 1376.69 | 26894.92 | 56212.30 | 123.84 |
| EELMKDIGDLSKK | 2 | 1504.78 | 1296.72 | 0.00 | 78.70 |
| SSREELMKDIGDLSK | 2 | 1706.85 | 31775.84 | 51143.30 | 126.88 |
| SSREELMKDIGDLSKK | 3 | 1834.95 | 1351.72 | 2290.26 | 68.75 |
| VLKDLQEK | 1 | 971.57 | 38204.13 | 44540.61 | 198.65 |
| WDAVDNK | 0 | 846.39 | 11545.73 | 3684.78 | 103.91 |
|  |  |  | 239561.8 | 341691.0 |  |
